# Supplementary material for: Prevalence of dengue in febrile patients in Peru: A systematic review and meta-analysis
Source: PLoS One. 2025 Jun 17;20(6):e0310163. doi: 10.1371/journal.pone.0310163 (PMC12173410; doi:10.1371/journal.pone.0310163)
Supplement: S2 Table — (DOCX) [file pone.0310163.s002.docx]

| S2 Table. The adjusted search terms as per searched electronic databases or search tools. | | | |
| --- | --- | --- | --- |
| PubMed | | | |
|  | #1 | ( "Febrile illness"[Title/Abstract] OR "febrile syndrome"[Title/Abstract] OR Dengue[Title/Abstract] OR "Breakbone Fever"[Title/Abstract] OR "Fever, Breakbone"[Title/Abstract] OR "Classical Dengue Fever"[Title/Abstract] OR "Break-Bone Fever"[Title/Abstract] OR "Break Bone Fever"[Title/Abstract] OR "Dengue Fever"[Title/Abstract] OR "Fever, Dengue"[Title/Abstract] OR "Classical Dengue"[Title/Abstract]) | 35,122 |
|  | #2 | Peru[Title] OR Peruvian[Title] | 9,329 |
|  | #3 | #1 AND #2 | 155 |
| Scopus | | | |
|  | #1 | TITLE( "Febrile illness" OR "febrile syndrome" OR Dengue OR "Breakbone Fever" OR "Classical Dengue Fever" OR "Break-Bone Fever" OR "Break Bone Fever" OR "Dengue Fever" OR "Classical Dengue") | 25,748 |
|  | #2 | TITLE(Peru OR Peruvian) | 32,971 |
|  | #3 | #1 AND #2 | 128 |
| Embase | | | |
|  | #1 | ("Febrile illness" OR 'dengue').ti,ab. | 44,465 |
|  | #2 | (' Peru' OR 'Peruvian').ti,ab. | 28,965 |
|  | #3 | #1 AND #2 | 152 |
| Web of Science | | | |
|  | #1 | TI=("Febrile illness" OR Dengue OR "Breakbone Fever" OR "Fever, Breakbone" OR "Classical Dengue Fever" OR "Break-Bone Fever" OR "Break Bone Fever" OR "Dengue Fever" OR "Fever, Dengue" OR "Classical Dengue") | 21,091 |
|  | #2 | TI=(Peru OR Peruvian) | 23,979 |
|  | #3 | #1 AND #2 | 118 |
| ScienceDirect | | | |
|  | #1 | Title, abstract, keywords: ( "Febrile illness" OR Dengue) | 9,574 |
|  | #2 | Title, abstract, keywords: ( Peru OR Peruvian ) | 8,760 |
|  | #3 | #1 AND #2 | 40 |
| Google Scholar | | |  |
|  | #1 | allintitle: "Febrile illness" OR Dengue" | 103,000 |
|  | #2 | allintitle: "Peru OR Peruvian" | 150,000 |
|  | #3 | #1 AND #2 | 530 |
| Virtual Health Library (VHL) | | | |
|  | #1 | ti:("Febrile illness" OR Dengue) | 26,417 |
|  | #2 | ti:( Peru OR Peruvian) | 19,905 |
|  | #3 | #1 AND #2 | 187 |
| Scielo | | | |
|  | #1 | ti:(*dengue OR "Febrile illness") | 2,735 |
|  | #2 | ti:(*Peru) | 12,568 |
|  | #3 | #1 AND #2 | 80 |
| TOTAL |  | PubMed (n =155); Scopus (n=128); Web of Sciences(n=118); Embase (n=152); ScienceDirect (40); Google Scholar (530); Virtual Health Library (187); and Scielo (80) | 1,390 |
| Duplicate records eliminated | | | 800 |
| Records examined | | | 590 |
| Records excluded by title and abstract | | | 536 |
| Reports evaluated by full text | | | 49 |
| Reports excluded by full text | | | 34 |
| Reports included in full text | | | 15 |
