# Supplementary material for: Prevalence of dengue in febrile patients in Peru: A systematic review and meta-analysis
Source: PLoS One. 2025 Jun 17;20(6):e0310163. doi: 10.1371/journal.pone.0310163 (PMC12173410; doi:10.1371/journal.pone.0310163)
Supplement: S3 Table — (DOCX) [file pone.0310163.s003.docx]

**S3 Table**. Quality of the dengue studies included in the review

| Authors | Year | Eligibility criteria | Study subjects and the setting | Exposure measured in a valid and reliable way 'gold standard' | A specified diagnosis or definition | Confounding factors | Dealing with confounding factors | Outcomes measured in a valid and reliable way | Appropriate statistical analysis | Scores (8) | Quality (high, moderate, low) |  |
| --- | --- | --- | --- | --- | --- | --- | --- | --- | --- | --- | --- | --- |
| Valdivia-Conroy B, et al. (1) | | 2022 | Yes | Yes | Yes | Yes | Unclear | NA | Yes | Yes | 6 | Moderate |
| arazona-Castro Y, et al. (2) | | 2022 | Yes | Yes | Yes | Yes | Unclear | NA | Yes | Yes | 6 | Moderate |
| Del Valle-Mendoza J, et al. (3) | | 2021 | Yes | Yes | Yes | Yes | Unclear | NA | Yes | Yes | 6 | Moderate |
| Aguilar-Luis MA, et al. (4) | | 2021 | Yes | Yes | Yes | Yes | Unclear | NA | Yes | Yes | 6 | Moderate |
| Elson WH,et al. (5) | | 2020 | Yes | Yes | Yes | Yes | Unclear | NA | Yes | Yes | 6 | Moderate |
| Del Valle-Mendoza J, et al. (6) | | 2020 | Yes | Yes | Yes | Yes | Unclear | NA | Yes | Yes | 6 | Moderate |
| Palomares-Reyes C, et al. (7) | | 2019 | Yes | Yes | Yes | Yes | Unclear | NA | Yes | Yes | 6 | Moderate |
| Torres – Coronado PE, et al. (8) | | 2019 | Yes | Yes | Yes | Yes | Unclear | NA | Yes | Yes | 6 | Moderate |
| Sánchez-Carbonel J, et al. (9) | | 2018 | Yes | Yes | Yes | Yes | Unclear | NA | Yes | Yes | 6 | Moderate |
| Alva-Urcia C, et al. (10) | | 2017 | Yes | Yes | Yes | Yes | Unclear | NA | Yes | Yes | 6 | Moderate |
| Loayza M, et al. (11) | | 2010 | Yes | Yes | Yes | Yes | Unclear | NA | Yes | Yes | 6 | Moderate |
| Troyes RL, et al. (12) | | 2006 | Yes | Yes | Yes | Yes | Unclear | NA | Yes | Yes | 6 | Moderate |
| Gómez B, et al. (13) | | 2005 | Yes | Yes | Yes | Yes | Unclear | NA | Yes | Yes | 6 | Moderate |
| Cobos Z, et al. (14) | | 2004 | Yes | Yes | Yes | Yes | Unclear | NA | Yes | Yes | 6 | Moderate |
| Mostorino ER, et al. (15) | | 2002 | Yes | Yes | Yes | Yes | Unclear | NA | Yes | Yes | 6 | Moderate |

NA: Not assessed

Moola S, Munn Z, Tufanaru C, Aromataris E, Sears K, Sfetcu R, Currie M, Qureshi R, Mattis P, Lisy K, Mu P-F Chapter 7: Systematic reviews of etiology and risk: JBI; 2020. Available from: <https://synthesismanual.jbi.global>

**Bibliographical references**

1. Valdivia-Conroy B, Vasquez-Calderón JM, Silva-Caso W, Martins-Luna J, Aguilar-Luis MA, Del Valle-Mendoza J, et al. Diagnostic performance of the rapid test for the detection of NS1 antigen and IgM and IgG anti-antibodies against dengue virus. Rev Peru Med Exp Salud Publica. 2022;39(4):434-41.

2. Tarazona-Castro Y, Troyes-Rivera L, Martins-Luna J, Cabellos-Altamirano F, Aguilar-Luis MA, Carrillo-Ng H, et al. Detection of SARS-CoV-2 antibodies in febrile patients from an endemic region of dengue and chikungunya in Peru. PloS One. 2022;17(4):e0265820.

3. J DVM, C PR, H CN, Y TC, S K, Ma AL, et al. Leptospirosis in febrile patients with suspected diagnosis of dengue fever. BMC Res Notes [Internet]. 29 de mayo de 2021 [citado 6 de julio de 2024];14(1). Disponible en: https://pubmed.ncbi.nlm.nih.gov/34051849/

4. Aguilar-Luis MA, Carrillo-Ng H, Kym S, Silva-Caso W, Verne E, Valle LJD, et al. Detection of Dengue Virus Serotype 3 in Cajamarca, Peru: Molecular Diagnosis and Clinical Characteristics. Int J Infect Dis. 1 de marzo de 2022;116:S119-20.

5. Elson WH, Reiner RC, Siles C, Bazan I, Vilcarromero S, Riley-Powell AR, et al. Heterogeneity of Dengue Illness in Community-Based Prospective Study, Iquitos, Peru. Emerg Infect Dis. septiembre de 2020;26(9):2077-86.

6. J DVM, F VA, Ma AL, J ML, J BM, V ZG, et al. Unidentified dengue serotypes in DENV positive samples and detection of other pathogens responsible for an acute febrile illness outbreak 2016 in Cajamarca, Peru. BMC Res Notes [Internet]. 10 de junio de 2020 [citado 6 de junio de 2024];13(1). Disponible en: https://pubmed.ncbi.nlm.nih.gov/33023645/

7. Palomares-Reyes C, Silva-Caso W, Del Valle LJ, Aguilar-Luis MA, Weilg C, Martins-Luna J, et al. Dengue diagnosis in an endemic area of Peru: Clinical characteristics and positive frequencies by RT-PCR and serology for NS1, IgM, and IgG. Int J Infect Dis IJID Off Publ Int Soc Infect Dis. abril de 2019;81:31-7.

8. Coronado PET, Aguilar-Gamboa FR, Guevara-Vásquez G. Características epidemiológicas y serológicas de los pacientes con dengue probable, en un hospital de Lambayeque, Perú: Epidemiological and serological characteristics of patients with probable dengue, in a Lambayeque hospital, Peru. Rev Exp En Med Hosp Reg Lambayeque. 15 de abril de 2019;5(1):13-7.

9. Sánchez-Carbonel J, Tantaléan-Yépez D, Aguilar-Luis MA, Silva-Caso W, Weilg P, Vásquez-Achaya F, et al. Identification of infection by Chikungunya, Zika, and Dengue in an area of the Peruvian coast. Molecular diagnosis and clinical characteristics. BMC Res Notes. 14 de marzo de 2018;11(1):175.

10. Alva-Urcia C, Aguilar-Luis MA, Palomares-Reyes C, Silva-Caso W, Suarez-Ognio L, Weilg P, et al. Emerging and reemerging arboviruses: A new threat in Eastern Peru. PloS One. 2017;12(11):e0187897.

11. Loayza M, Cisneros GA, Loro L, Yale G. Dengue fever outbreak in Lima, Peru 2009: Epidemiological changes in urban areas. Int J Infect Dis. 1 de marzo de 2010;14:e177.

12. Troyes R L, Fuentes T L, Troyes R M, Canelo D L, García M M, Anaya R E, et al. Etiología del síndrome febril agudo en la provincia de Jaén, Perú 2004-2005. Rev Peru Med Exp Salud Publica. enero de 2006;23(1):5-11.

13. Gómez B J, Mostorino E R, Chinchay M R, García M M, Roldán A L, Ruiz O J. Seroprevalencia del dengue en el distrito de Casma. Ancash, Perú 2002. Rev Peru Med Exp Salud Publica. 2005;200-4.

14. Z MC, P VG, M MG, Z EM, C RF, D RR, et al. Estudio serológico y virológico del brote de dengue en la provincia de Coronel Portillo. Ucayali, Perú (2000 - 2001). Rev Peru Med Exp Salud Pública [Internet]. 1 de enero de 2004 [citado 6 de julio de 2024]; Disponible en: https://rpmesp.ins.gob.pe/index.php/rpmesp/article/view/950

15. Mostorino E R, Rosas A Á, Gutiérrez P V, Anaya R E, Cobos M, García M M. Manifestaciones Clínicas y Distribución Geográfica de los Serotipos del Dengue en el Perú - Año 2001. Rev Peru Med Exp Salud Publica. octubre de 2002;19(4):171-80.
