# Supplementary material for: Prevalence of dengue in febrile patients in Peru: A systematic review and meta-analysis
Source: PLoS One. 2025 Jun 17;20(6):e0310163. doi: 10.1371/journal.pone.0310163 (PMC12173410; doi:10.1371/journal.pone.0310163)
Supplement: S5 Table — (DOCX) [file pone.0310163.s005.docx]

**S5 Table**. Meta-analysis database

| **Prevalence of dengue using ELISA IgG** | | | |
| --- | --- | --- | --- |
| **Study** | **year** | **Sample** | **IgG** |
| **Valdivia-Conroy B, et al.** | 2022 | 286 | 156 |
| **Aguilar-Luis MA, et al.** | 2021 | 359 | 60 |
| **Palomares-Reyes C, et al.** | 2019 | 268 | 42 |
| **Torres – Coronado PE, et al.** | 2019 | 709 | 61 |
| **Gómez B, et al.** | 2005 | 400 | 61 |
| **Prevalence of dengue using ELISA IgM** | | | |
| **Study** | **year** | **Sample** | **IgM** |
| **Valdivia-Conroy B, et al.** | 2022 | 286 | 54 |
| **arazona-Castro Y, et al.** | 2022 | 464 | 43 |
| **Aguilar-Luis MA, et al.** | 2021 | 359 | 35 |
| **Palomares-Reyes C, et al.** | 2019 | 268 | 28 |
| **Torres – Coronado PE, et al.** | 2019 | 709 | 136 |
| **Loayza M, et al.** | 2010 | 552 | 148 |
| **Troyes RL, et al.** | 2006 | 1039 | 105 |
| **Gómez B, et al.** | 2005 | 400 | 40 |
| **Cobos Z, et al.** | 2004 | 742 | 142 |
| **Mostorino ER, et al.** | 2002 | 6072 | 1593 |
| **Prevalence of dengue using PCR (ARN)** | | | |
| **Study** | **year** | **Sample** | **ARN** |
| **arazona-Castro Y, et al.** | 2022 | 464 | 21 |
| **Del Valle-Mendoza J, et al.** | 2021 | 276 | 84 |
| **Aguilar-Luis MA, et al.** | 2021 | 359 | 89 |
| **Elson WH,et al.** | 2020 | 429 | 79 |
| **Del Valle-Mendoza J, et al.** | 2020 | 124 | 32 |
| **Palomares-Reyes C, et al.** | 2019 | 268 | 69 |
| **Sánchez-Carbonel J, et al.** | 2018 | 496 | 170 |
| **Alva-Urcia C, et al.** | 2017 | 139 | 9 |
| **Loayza M, et al.** | 2010 | 552 | 99 |
| **Prevalence of dengue using ELISA NS1** | | | |
| **Study** | **year** | **Sample** | **NS1** |
| **Valdivia-Conroy B, et al.** | 2022 | 286 | 97 |
| **arazona-Castro Y, et al.** | 2022 | 464 | 21 |
| **Aguilar-Luis MA, et al.** | 2021 | 359 | 109 |
| **Palomares-Reyes C, et al.** | 2019 | 268 | 51 |
| **Torres – Coronado PE, et al.** | 2019 | 709 | 108 |
