# Supplementary material for: Prevalence of dengue in febrile patients in Peru: A systematic review and meta-analysis
Source: PLoS One. 2025 Jun 17;20(6):e0310163. doi: 10.1371/journal.pone.0310163 (PMC12173410; doi:10.1371/journal.pone.0310163)
Supplement: S6 Table — (DOCX) [file pone.0310163.s006.docx]

**S6 Table**. R version 4.2.3. script

| **Import database** |
| --- |
| Environment – From Excel – Import Excel Data - Import |
| **Calculate the prevalence of dengue using ELISA IgG.** |
| library(meta)  library(readxl)  data_prevalence<- **Database name**  mtprop=metaprop(event= **IgG, IgM, ARN or NS1**,  n= **Sample_** **Study**,  studlab=paste(Study,year),  data=data_prevalence,  method.tau = "DL",  method = "Inverse",  method.ci = "SACC",  sm="PFT")  mtprop  forest(mtprop,  comb.fixed=FALSE,  common = FALSE)  **An example is given**:  Database name: **Base_IgG**   \| Study \| year \| **Sample** \| **IgG** \| \| --- \| --- \| --- \| --- \| \| **Valdivia-Conroy B, et al.** \| 2022 \| 286 \| 156 \| \| **Aguilar-Luis MA, et al.** \| 2021 \| 359 \| 60 \| \| **Palomares-Reyes C, et al.** \| 2019 \| 268 \| 42 \| \| **Torres – Coronado PE, et al.** \| 2019 \| 709 \| 61 \| \| **Gómez B, et al.** \| 2005 \| 400 \| 61 \|   **Code in R version 4.2.3**  library(meta)  library(readxl)  data_prevalence<- **Base_IgG**  mtprop=metaprop(event= **IgG**,  n= **Sample**,  studlab=paste(Study,year),  data=data_prevalence,  method.tau = "DL",  method = "Inverse",  method.ci = "SACC",  sm="PFT")  mtprop  forest(mtprop,  comb.fixed=FALSE,  common = FALSE)  **Results**  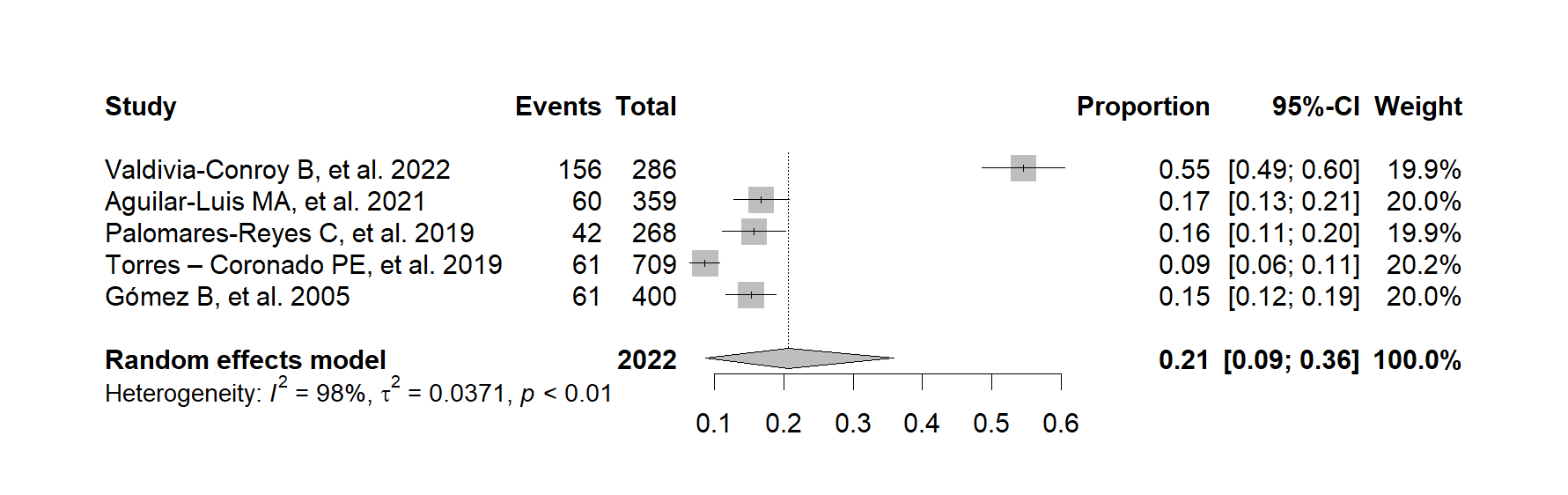 |
| **Funnel plot and Egger's test** |
| library(meta)  library(readxl)  par(mar = c(5, 5, 2, 2))  data_prevalence<- **Base_IgM**  mtprop=metaprop(event= **IgM**,  n= **Sample**,  studlab=paste(Study,year),  data=data_prevalence,  method.tau = "DL",  method = "Inverse",  method.ci = "SACC",  sm="PFT")  par(mar = c(5, 5, 2, 2))  funnel(mtprop, comb.fixed = FALSE, common = FALSE)  par(mar = c(5, 5, 2, 2))  egger_test <- metabias(mtprop)  print(egger_test)  **An example is given**:  Database name: **Base_IgM**   \| Study \| year \| **Sample** \| **IgM** \| \| --- \| --- \| --- \| --- \| \| **Valdivia-Conroy B, et al.** \| 2022 \| 286 \| 54 \| \| **arazona-Castro Y, et al.** \| 2022 \| 464 \| 43 \| \| **Aguilar-Luis MA, et al.** \| 2021 \| 359 \| 35 \| \| **Palomares-Reyes C, et al.** \| 2019 \| 268 \| 28 \| \| **Torres – Coronado PE, et al.** \| 2019 \| 709 \| 136 \| \| **Loayza M, et al.** \| 2010 \| 552 \| 148 \| \| **Troyes RL, et al.** \| 2006 \| 1039 \| 105 \| \| **Gómez B, et al.** \| 2005 \| 400 \| 40 \| \| **Cobos Z, et al.** \| 2004 \| 742 \| 142 \| \| **Mostorino ER, et al.** \| 2002 \| 6072 \| 1593 \|   **Results**  **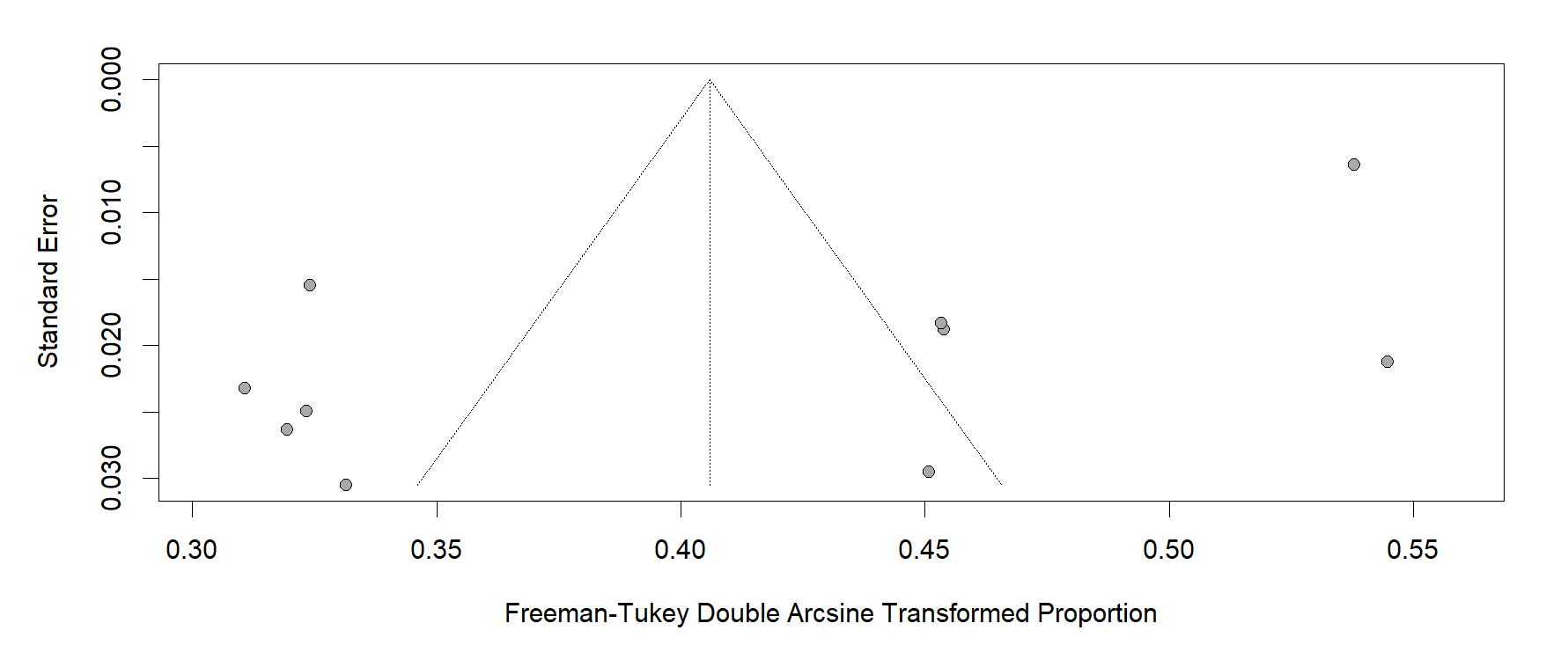** |
