# Supplementary figures and images for: Prevalence of dengue in febrile patients in Peru: A systematic review and meta-analysis
Source: PLoS One. 2025 Jun 17;20(6):e0310163. doi: 10.1371/journal.pone.0310163 (PMC12173410; doi:10.1371/journal.pone.0310163)

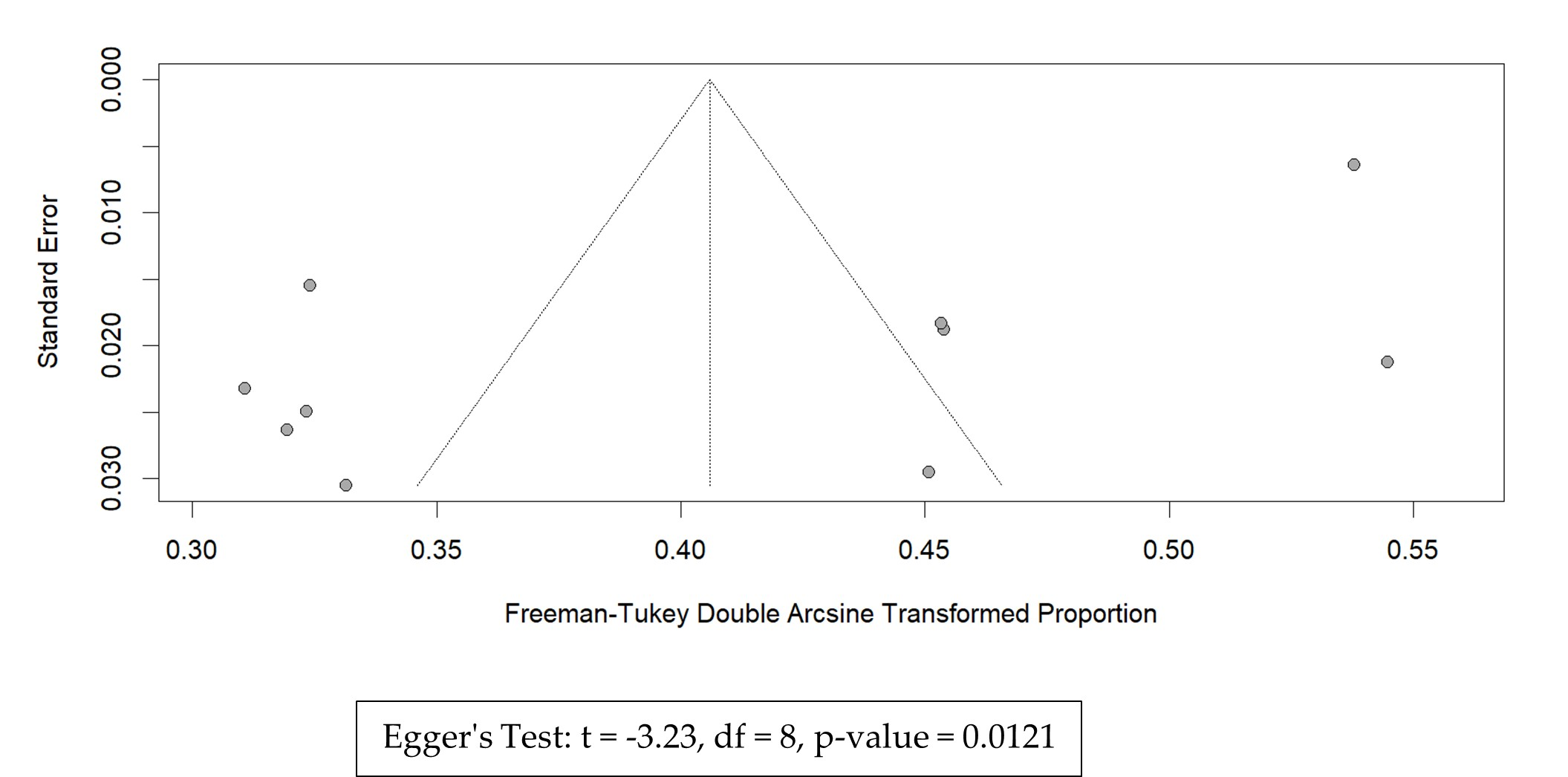

Supplement: S1 Fig — (TIF) [file pone.0310163.s007.tif]
