# Supplementary material for: Prevalence of dengue in febrile patients in Peru: A systematic review and meta-analysis
Source: PLoS One. 2025 Jun 17;20(6):e0310163. doi: 10.1371/journal.pone.0310163 (PMC12173410; doi:10.1371/journal.pone.0310163)
Supplement: Attachment — Submitted filename: Response to Editor.pdf [file pone.0310163.s009.pdf]

Editor's comments

1. **Thank you for addressing most of our recent requests. However, the following item still appears to be outstanding:**
   As required by our policy on Data Availability, please ensure your manuscript or supplementary information includes the following:

Our response: Thank you very much for your comments. I will respond to your inquiry below.

2. **1.) A numbered table of all studies identified in the literature search, including those that were excluded from the analyses.**

Our response: A table of the electronic search and a table of the excluded studies with the relevant reasons have been presented in detail. Review the tables of supplementary material (**S2 Table and S4 Table**).

	#2	ti:(Peru OR Peruvian)	19,905
	#3	#1 AND #2	187
**Scielo**			
	#1	ti:(*dengue OR "Febrile illness")	2,735
	#2	ti:(*Peru)	12,568
	#3	#1 AND #2	80
**TOTAL**		PubMed (n =155); Scopus (n=128); Web of Sciences(n=118); Embase (n=152); ScienceDirect (40); Google Scholar (530); Virtual Health Library (187); and Scielo (80)	1,390
**Duplicate records eliminated**			800
**Records examined**			590
**Records excluded by title and abstract**			536
**Reports evaluated by full text**			49
**Reports excluded by full text**			34
**Reports included in full text**			15

a. **For every excluded study, the table should list the reason(s) for exclusion.**

Our response: The reasons for exclusion of studies are listed in the Supplementary Material **S4 Table**. Please review the supplementary material documents.

B593

		F	G	H	I
1	s	url	doi	Reason for exclusion and inclusion	
2	ınd Suц‖rez, V. and Being		10.1371/journal.pntd.0000514	Records Excluded- Title/Abstract Screening: irrelevant to the research question	
3	:. and Rocha, C. and Watt		10.4269/ajtmh.2010.10-0116	Records Excluded- Title/Abstract Screening: irrelevant to the research question	
4	and Turell, M. J. and O'Gu			Records Excluded- Title/Abstract Screening: irrelevant to the research question	
5	e-Mendoza, J. and Silva-C		10.1016/j.ijid.2020.01.024	Records Excluded- Title/Abstract Screening: irrelevant to the research question	
6	Luna, J. and Silva-Caso, \			Records Excluded- Title/Abstract Screening: irrelevant to the research question	
7	ıbé-Castillo, R. V. and All	https://www.scopus.com/inward/rec	10.15381/anales.v83i4.23468	Records Excluded- Title/Abstract Screening: irrelevant to the research question	
8	ti, Sulistyawati	https://app.dimensions.ai/details/pu	10.11591/ijphs.v13i4.24454	Records Excluded- Title/Abstract Screening: irrelevant to the research question	
9	d Diego, Azañedo and Sta	https://app.dimensions.ai/details/pu	10.21149/7885	Records Excluded- Title/Abstract Screening: irrelevant to the research question	
10	edro María, Alarcón-Elbal	https://app.dimensions.ai/details/pu	10.31005/iajmh.v7i.261	Records Excluded- Title/Abstract Screening: irrelevant to the research question	
11	Dario, Spiller and Raquel,	https://app.dimensions.ai/details/pu	10.1038/s41598-024-52796-9	Records Excluded- Title/Abstract Screening: irrelevant to the research question	
12	r, Falcón	https://app.dimensions.ai/details/pu	10.15381/rivep.v32i2.20005	Records Excluded- Title/Abstract Screening: irrelevant to the research question	
13		https://app.dimensions.ai/details/pu	10.31243/id.v12.2020.992	Records Excluded- Title/Abstract Screening: irrelevant to the research question	
14	is Amin, Blanco and Luis I	https://app.dimensions.ai/details/pu		Records Excluded- Title/Abstract Screening: irrelevant to the research question	
15	id, Romero and Kris, Murr	https://app.dimensions.ai/details/pu	10.1111/ecog.06942	Records Excluded- Title/Abstract Screening: irrelevant to the research question	
16	omayor, Ureta and Jesus	https://app.dimensions.ai/details/pu	10.52808/bmsa.7e6.624.029	Records Excluded- Title/Abstract Screening: irrelevant to the research question	
17	Carlos Ramiro, Silva-Rar	https://app.dimensions.ai/details/pu	10.1186/s13071-022-05289-5	Records Excluded- Title/Abstract Screening: irrelevant to the research question	
18		https://app.dimensions.ai/details/pu	10.1093/aepp/ppy027	Records Excluded- Title/Abstract Screening: irrelevant to the research question	
19	nd Patricia Matilde Huall	https://app.dimensions.ai/details/pu	10.52808/bmsa.8e7.63ee.011	Records Excluded- Title/Abstract Screening: irrelevant to the research question	

Articles evaluated (n = 590)

b. If any of the included studies are unpublished, include a link (URL) to the primary source or detailed information about how the content can be accessed.:

Our response: Each excluded study with the reasons stated in the S4 table.

3. **You have included the 49 articles you conducted a full-text assessment of, but the total number of studies you identified for screening was 590. Please can you update your table to include all 590 papers.**

Our response: Dear Editor: We appreciate your comments. We have downloaded the 590 articles evaluated in Rayyan and organized the information in an Excel table, where we specify the articles evaluated by title, abstract, and full text. In addition, we detail the reasons for inclusion and exclusion in each article. In the supplementary material, we present the complete search and evaluation of the 590 articles, along with the corresponding reasons.

If you have any comments or recommendations, we are ready to respond.

Sincerely,

Mario J. Valladares-Garrido

Universidad Continental, Lima 15046, Peru; mvalladares@continental.edu.pe

Dear **Editor**,

Thank you very much for reviewing our article, "Prevalence of dengue in Peruvian patients with febrile illness according to RT-PCR and ELISA NS1, IgM, and IgG diagnostic tools: a systematic review and meta-analysis". Your suggestions and comments will be addressed below. Thank you for your valuable time and excellent review.

Editor's comments

1. **We note that your Data Availability Statement is currently as follows: "All relevant data are within the manuscript and its Supporting Information files."**

   Please confirm at this time whether or not your submission contains all raw data required to replicate the results of your study. Authors must share the "minimal data set" for their submission. PLOS defines the minimal data set to consist of the data required to replicate all study findings reported in the article, as well as related metadata and methods (https://journals.plos.org/plosone/s/data-availability#loc-minimal-data-set-definition).

Our response: *I confirm that all data are included in the manuscript and supplementary material.* Our study details all the steps and procedures necessary to replicate the findings reported in the article, along with the corresponding metadata and methods.
It was indicated according to the platform on which the data can be found in complete sentences.

Describe where the data may be found in full sentences. If you are copying our sample text, replace any instances of XXX with the appropriate details.

- If the data are **held or will be held in a public repository**, include URLs, accession numbers or DOIs. If this information will only be available after acceptance, indicate this by ticking the box below. For example: *All XXX files are available from the XXX database (accession number(s) XXX, XXX.*).
- If the data are all contained **within the manuscript and/or Supporting Information files**, enter the following: *All relevant data are within the manuscript and its Supporting Information files.*
- If neither of these applies but you are able to provide **details of access elsewhere**, with or without limitations, please do so. For example:

  Data cannot be shared publicly because of [XXX]. Data are available from the XXX Institutional Data Access / Ethics Committee (contact via XXX) for researchers who meet the criteria for access to confidential data.

  The data underlying the results presented in the study are available from (include the name of the third party and contact information or URL).

2. **As required by our policy on Data Availability, please ensure your manuscript or supplementary information includes the following:**

Our response: Supplementary information for our systematic review includes all documents that support the entire research process. In addition, each document follows the format required by PLOS ONE and is detailed in the article's supplementary material files.

Supporting information

S1 Table. PRISMA Checklist (PRISMA 2020 Main Checklist and PRIMSA Abstract Checklist).

S2 Table. The adjusted search terms as per searched electronic databases or search tools.

S3 Table. Quality of the studies included in the systematic review and meta-analysis.

S4 Table. Table of excluded studies.

S5 Table. Meta-analysis database

S6 Table. R version 4.2.3. script.

S1 Fig. The funnel plot and Egger's test illustrate the publication bias of the included studies assessing the pooled prevalence of dengue in Peruvian patients with febrile illness by IgM ELISA.

If you have any comments or recommendations, we are ready to respond.

Sincerely,

Mario J. Valladares-Garrido

Universidad Continental, Lima 15046, Peru; mvalladares@continental.edu.pe

Dear **Editor**,

Thank you very much for reviewing our article, "Prevalence of dengue in Peruvian patients with febrile illness according to RT-PCR and ELISA NS1, IgM, and IgG diagnostic tools: a systematic review and meta-analysis". Your suggestions and comments will be addressed below. Thank you for your valuable time and excellent review.

Editor's comments

1. Your manuscript is a wonderful review of Dengue diagnostics in Peru. However, both reviewers have pointed out areas of your paper that need clarification and explanation to improve the relevance of your findings.

   Our response: Thank you very much for the valuable comments and recommendations from the reviewers and from you, dear editor. Each comment has been carefully reviewed and a detailed response, including relevant clarifications and explanations, has been provided.

2. I agree with Reviewer 2 that google scholar is not a data base and use of this should be defined. For myself, I am curious why you did not include LILACS database as part of your search as it is specific to Latin America and often identifies studies and reports not found in the databases used in your manuscript.

   Our response: We agree with the reviewers' observation that Google Scholar is a search engine rather than a database. Accordingly, we have reviewed other meta-analyses that have been published in Plos One and have applied the relevant changes in the methodology and abstract sections, as per their request.

   - https://doi.org/10.1371/journal.pone.0311110
   - https://doi.org/10.1371/journal.pone.0310405
   - https://doi.org/10.1371/journal.pone.0309692
   - https://doi.org/10.1371/journal.pone.0308419

   The LILACS database was not included in the search since the articles belong to the Virtual Health Library, an important regional database for Latin America.

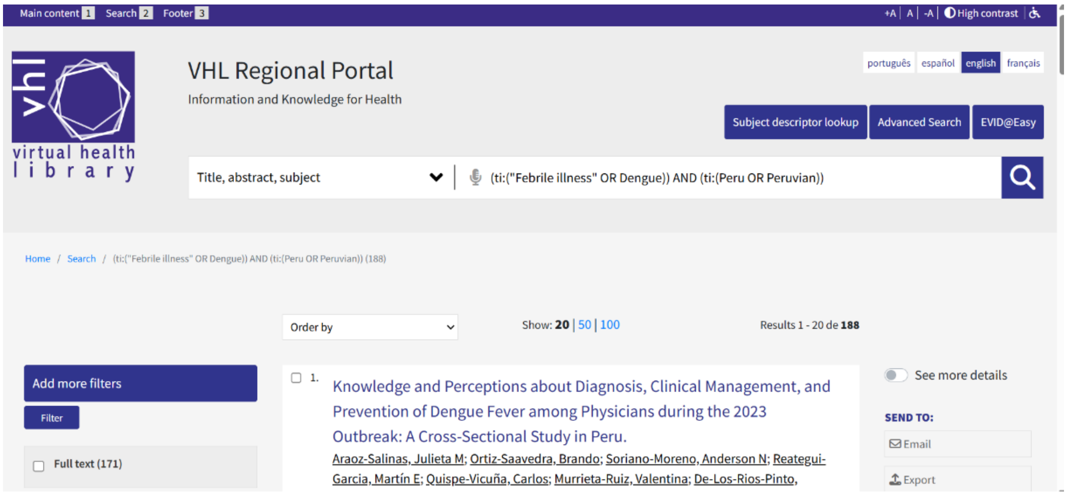

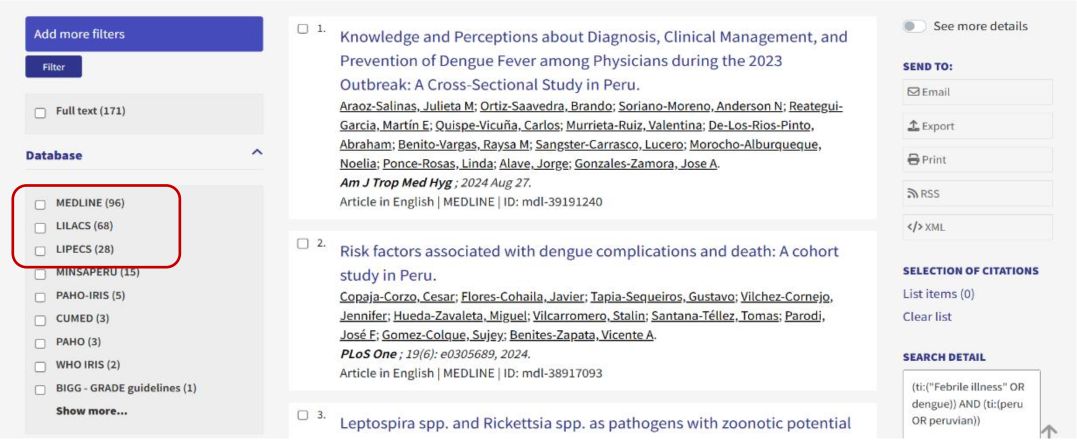

3. I look forward to reading your revised manuscript.

   Our response: We sincerely appreciate their comments. All comments made by the reviewers have been addressed, and the corresponding modifications have been implemented in accordance with their recommendations.

Reviewer #1:

Prevalence of dengue in Peruvian patients with febrile illness according to RT-PCR and ELISA NS1, IgM, and IgG diagnostic tools: systematic review and meta-analysis.
In my opinion, the manuscript can be published if the following observations are clarified or raised:
Our response: "We sincerely thank you for your comments and recommendations, which have contributed significantly to improving the quality of our article. The corresponding modifications have been made to the text, and a detailed response has been provided to each of the comments."

Summary section:

1. **Reviewer says:** "1. In the methodology section, indicate only the databases used: Google Scholar is not a database, for example."
   Our response: "We agree with your observation that Google Scholar is not a database but a search tool. Therefore, we have made the corresponding modifications in the abstract and methodology sections, following your recommendation and reviewing other meta-analyses published in Plos One, and The Lancet."
   - https://doi.org/10.1371/journal.pone.0311110
   - https://doi.org/10.1371/journal.pone.0310405
   - https://doi.org/10.1371/journal.pone.0309692
   - https://doi.org/10.1371/journal.pone.0308419

2. **Reviewer says:** "2. Indicate that observational studies with a control group were considered."
   Our response: "The aforementioned recommendation was incorporated, highlighting that the study included observational studies with a control group, which allowed comparing patients diagnosed with dengue and those with other causes of fever according to the established inclusion criteria. This facilitated a more precise evaluation of the effectiveness of diagnostic tools, such as RT-PCR and ELISA NS1, IgM, and IgG.

3. **Reviewer says:** “3. The keywords must be Mesh terms.”
Our response: “In the summary and methodology section, the use of MeSH terms was corrected, ensuring their correct application. In addition, we specified the use of a free term of relevance in the search strategy, the importance of which was verified through published studies.”

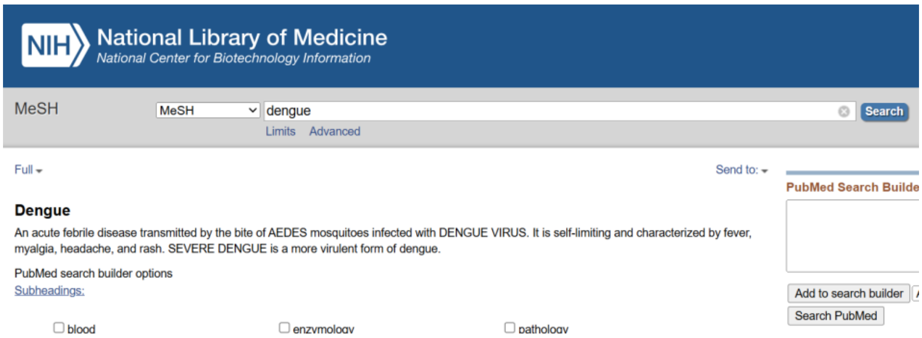

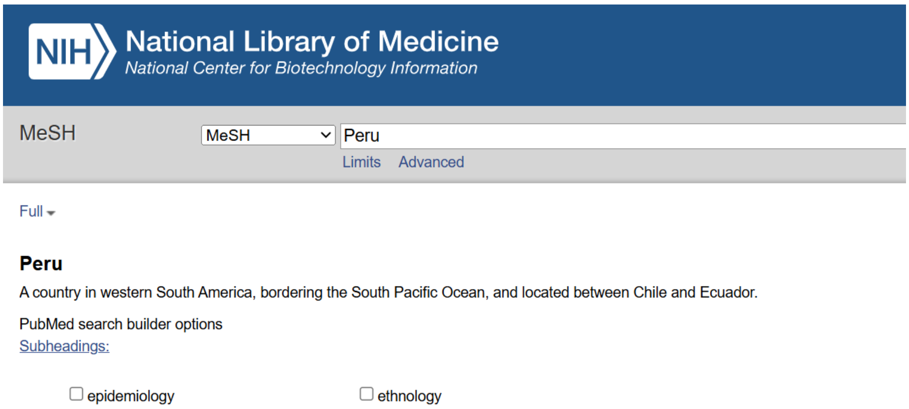

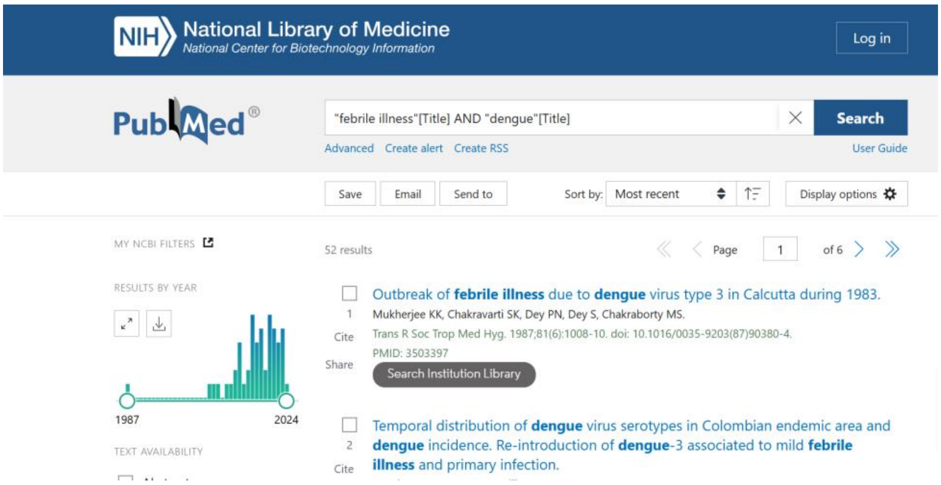

Introduction section:

4. **Reviewer says:** "4. This section should refer to other systematic reviews with or without meta-analysis on the research question carried out in Peru or Latin America.
Our response: "In accordance with the suggestion provided, we searched for systematic reviews, with or without meta-analysis, related to the research question in Peru and Latin America. The results of these reviews have been incorporated in the introduction section to contextualize and strengthen the theoretical framework of the study."

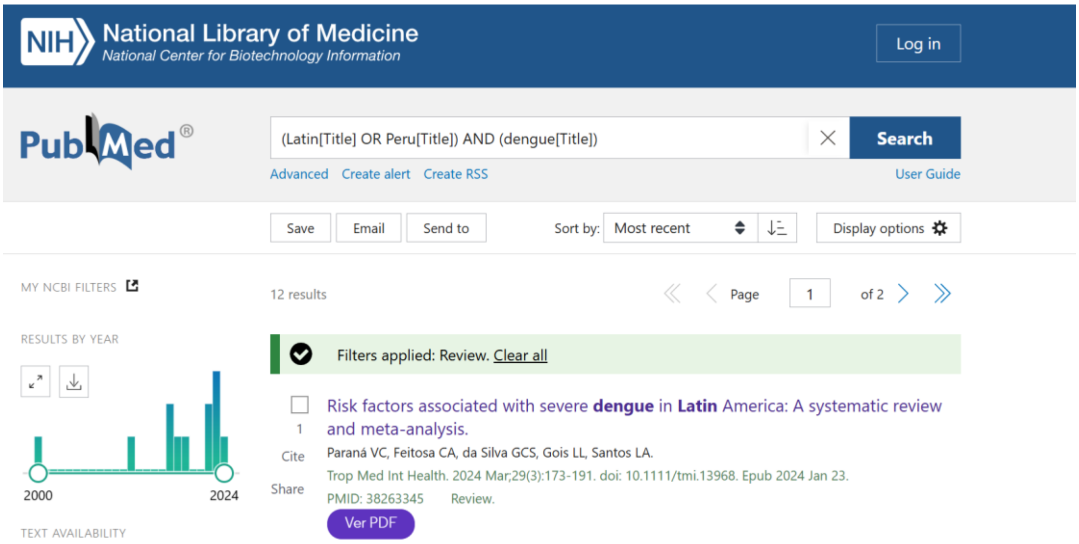

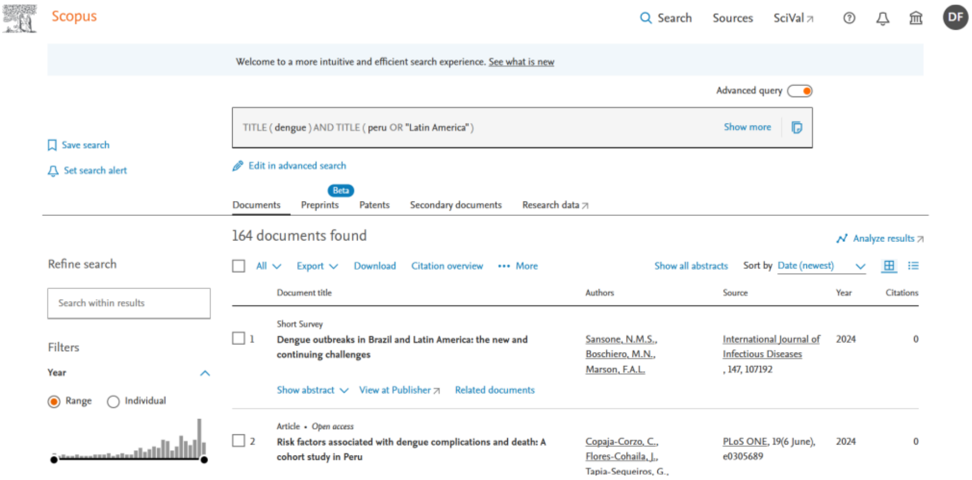

<u>Methodology section:</u>

5. **Reviewer says:** "5. Indicate whether there were deviations from the original protocol registered in PROSPERO."
Our response: "The methodology section mentioned that the original protocol registered in PROSPERO underwent some minor adjustments. These changes, which included the inclusion of studies with and without a control group, the updating of the team of authors, and the incorporation of more researchers for the evaluation, selection of articles, and data analysis, were also detailed."

6. **Reviewer says:** "6. It is necessary to indicate that observational studies without a control group were considered and for what reason?"

Our response: "The recommendation was incorporated, emphasizing the inclusion of observational studies without a control group. This decision was based on the nature of the research problem, since many epidemiological studies on the prevalence of dengue in Peru do not have a control group due to contextual or practical limitations. Despite this absence, such studies provide relevant information on the prevalence of dengue in febrile patients, enriching the understanding of incidence and allowing a broader analysis of the diagnostic tools used. Recognizing the limitations of the lack of control, we consider that their inclusion significantly broadens the available evidence base.

7. **Reviewer says:** "7. Again, Google Scholar is not a database; these sources or information must be changed."
Our response: We agree with the reviewers' observation that Google Scholar is a search engine rather than a database. Accordingly, we have reviewed other meta-analyses that have been published in Plos One and have applied the relevant changes in the methodology and abstract sections, as per their request.
    - https://doi.org/10.1371/journal.pone.0311110
    - https://doi.org/10.1371/journal.pone.0310405
    - https://doi.org/10.1371/journal.pone.0309692
    - https://doi.org/10.1371/journal.pone.0308419

8. **Reviewer says:** "8. State the limitations of the bias risk assessment tool for the studies considered. Considering the following points: Is this tool relevant? Does it assess the ability to develop a meta-analysis from the studies? Does it assess heterogeneity?"
Our response: "The recommendation was added. In addition, the tool used was clarified. Although the JBI-MAStARI tool is useful for assessing bias in individual studies, it has important limitations, as it does not directly assess the feasibility of performing a meta-analysis nor does it address heterogeneity between studies. To obtain a more complete assessment in the context of a meta-analysis, we employed additional methods that considered both publication bias and heterogeneity."

Limitations section

9. **Reviewer says:** "9. Add the limitations of the work. Mainly the risk of bias after analyzing the studies.

Our response: "The suggestion provided was corrected and added. "
We also identified a possible publication bias, as evidenced by Egger's test (p = 0.0121) and the asymmetry observed in the funnel plots. This suggests that studies with negative or nonsignificant results may have been less likely to be published, which could have influenced our prevalence estimates. In addition, although rigorous inclusion criteria were applied, heterogeneity among studies in terms of design, sample size, and methodology could have affected the validity of the results.

Reviewer #2:

The Authors present a meta-analysis on the prevalence of dengue among febrile patients in Peru. It is a well-structured and well carried out study. They address the importance of Dengue as a reemerging infection worldwide with a heavy burden in Peru and the Americas. The methodology and results are clearly presented, and support the authors conclusions.

Our response: "We sincerely appreciate your comments and suggestions, which have greatly improved the quality of our research. The text has been modified according to the comments, and each of them has been dealt with in detail."

The authors mention limitations for the diagnosis of dengue given the technological and infrastructural requirements for dengue diagnosis in poor communities but decided not to include lateral flow test/Rapid test in their study.

Our response: "Thank you very much for your observation. Regarding the exclusion of a more accessible diagnostic tool, such as the rapid test, when mentioning the technological difficulties in the areas studied, the tests used in this study for the diagnosis of dengue are based on what is established by the clinical practice guide of the Peruvian Ministry of Health. For this reason, both in the limitations and strengths sections, reference is made to the tests used."

The authors fail to address the limitation of determining prevalence of an acute infection in a febrile patient through a serological test, were due to exposures to many pathogens' antibodies might be secondary to previous infections and the necessarily the current cause of fever.

Our response: "Thank you for your valuable suggestion. We have proceeded to make the corresponding modifications and additions according to your recommendation.

"In addition, we recognize that determining the prevalence of an acute infection in a febrile patient by serological testing has limitations, as the antibodies detected could be the result of previous exposures to other pathogens and not necessarily the current cause of the fever. For this reason, various diagnostic tests were employed in order to obtain a more accurate picture of the current clinical situation."

Overall, I believe this is a good and valuable manuscript and should be considered for publication following minor changes.

Our response: "Thank you very much for your valuable comments and recommendations. Each of them has been carefully addressed and reflected in the article.

<u>Specific areas for improvement</u>

1. **Reviewer says:** "In the methods section of the abstract the authors state that "Study selection, quality assessment, and data extraction were performed independently by more than two authors" however in the study protocol it says this was done by two others with a third author only coming in to solve disagreements."
   Our response: "We agree with the observation related to the protocol registered in PROSPERO. However, the summary concisely reflects what is specified in the methodology of the article. We reiterate that there were slight variations in the protocol, mainly due to the incorporation of more researchers in our multidisciplinary team. To ensure greater rigor in the research, three authors were assigned the tasks of selection, quality assessment, and data extraction."
   The current investigation followed the Preferred Reporting Items for Systematic reviews and Meta-Analyses (PRISMA) guidelines (S1 Table) as well as a protocol registered in the Prospective International Registry of Systematic Reviews (PROSPERO) with the number CRD42024558891.The original protocol registered in PROSPERO underwent some minor

adjustments. These included specifying the inclusion of both studies with and without a control group, updating the team of authors who collaborated in the study, and the incorporation of more researchers in charge of evaluation, article selection, and data analysis.

2. **Reviewer says:** "In the Results section of the Abstract why are there to different results for IgG Elisa the authors might want to clarify the difference between one and the other.
   Our response: "Thank you very much for the comment. The error has been corrected, as there was a duplicate transcription of the same sentence.

3. **Reviewer says:** "In the introduction line 4, page 3, the authors might consider rephrasing "a rise in body temperature of at least 38.0°C", as it could imply an increment of 38 on top of the persons normal temperature.
   Our response: "It was corrected according to your recommendation."
   In this context, the term 'fever' is commonly used as a synonym for 'febrile illness', which is defined as a body temperature reaching or exceeding 38.0°C.

4. **Reviewer says:** "In the introduction, line 1 of paragraph 2, the authors might consider rephasing the sentence as "…dengue virus, a positive-stranded RNA…", it might be better to have the word virus after RNA instead of after dengue or use virus after both dengue and RNA. It also seems a little redundant to say a virus causes an infectious disease.
   Our response: "It was corrected according to your recommendation."
   Aedes mosquitoes transmit the dengue virus, a positive-stranded RNA virus, which causes dengue.

5. **Reviewer says:** "In the first sentence of paragraph 3 of the introduction the authors might consider rephrasing and "asymptomatic symptoms" is not the best wording.
   Our response: "It was corrected according to your recommendation."
   Most people infected with dengue present with mild or no symptoms, which usually last between 2 and 7 days.

6. **Reviewer says:** "In the first sentence of paragraph 3 of the introduction the authors might consider rephrasing as malaria is not a viral disease and leptospirosis is a zoonosis not a vector borne disease
   Our response: "It was corrected according to your recommendation."
   A crucial aspect for the effective management of dengue is accurate and timely diagnosis, as its non-specific clinical manifestations can be similar to those of other mosquito-borne viral diseases such as yellow fever, chikungunya, and Zika, as well as other diseases like malaria, trypanosomiasis, leptospirosis, and Chagas disease.

7. **Reviewer says:** "The authors might want to reconsider their wording in the third sentence of paragraph four of the introduction as IgM and IgG antibodies can help differentiate between recent and past exposure not primary or secondary infection.
   Our response: "It was corrected according to your recommendation."
   In addition, indirect methods, such as detection of IgM and IgG antibodies by enzyme-linked immunosorbent assays (ELISA), are used to distinguish between recent and past

exposure to the virus. Past exposures may increase the risk of developing severe forms of the disease.

8. **Reviewer says:** "In the last part of paragraph 4 the authors might want to develop that idea, instead of talking about secondary infection, focus on how disease severity might increase when infection from some serovars occur in patients with previous infections by other serovars.
Our response: "It was corrected according to your recommendation."
Throughout their lives, people can become infected with different serotypes of dengue virus. Patients who have already been infected by one serotype and then contract another often develop more severe disease due to the antibody-dependent enhancement phenomenon, in which antibodies generated by the first infection facilitate replication of the new virus [19]. A meta-analysis revealed that, in Southeast Asia, the DENV-3 serotype was responsible for the highest percentage of severe cases in primary infections (people who had not previously had dengue). For secondary infections (people who become re-infected with a different serotype), serotypes DENV-2, DENV-3, and DENV-4 in Southeast Asia and DENV-2 and DENV-3 in other regions were associated with the highest percentages of severe cases [20].

9. **Reviewer says:** "In the last part of paragraph 5 the authors might consider their wording as them seem to want to address cross reactivity with other flavivirus but the instead mention "secondary flavivirus infections", this seems to insinuate coinfection.
Our response: "It was corrected according to your recommendation."
The information that generated confusion was eliminated.

10. **Reviewer says:** "The study selection process included in the methods differs from the one in the study protocol, the protocol states two researchers reviewed titles and abstracts with a third one coming in to help solve disagreements, while the methods state that 3 researchers reviewed titles and abstracts with a fourth one coming in to resolve disagreements.
Our response: "It was corrected according to your recommendation."
To ensure greater rigor in the research, three authors were assigned the tasks of study selection, study quality assessment, and data extraction. This division of labor ensures that the researchers participate more rigorously and meticulously in each phase of the research process.

11. **Reviewer says:** "The authors should review their explanation on how they assessed heterogeneity between studies, as the current explanation does not account for how a study between 50 and 75% would be categorized.
Our response: "It was corrected according to your recommendation."
To assess between-study variability, we applied the Cochrane Q statistic. Between-study heterogeneity was measured using the inconsistency index (I^2), classifying it as follows: low if the I^2 value was less than 25%, moderate if it was between 25% and 50%, and high if it exceeded 75%. I^2 values between 50% and 75% were considered indicative of substantial heterogeneity.

12. **Reviewer says:** "The authors need to address the limitation of determining prevalence of dengue in a febrile patient from an endemic community using IgG and to a lesser extent IgM.
Our response: "It was corrected according to your recommendation."

In addition, we recognize the limitations of serologic testing to determine the prevalence of acute infection in febrile patients, especially in dengue-endemic communities. The detection of antibodies, such as IgG and IgM, may reflect past exposures to other pathogens, or in the case of IgG, previous dengue infections, which complicates the interpretation of results and may not represent the current cause of fever. To minimize these limitations and to obtain a more accurate picture of the current clinical situation, a variety of complementary diagnostic tests were employed.

13. **Reviewer says:** "Talking about the implication of their findings, the authors mention a greater need for diagnostic tools, but fail to connect this to their findings or to suggest what test they recommend being used based on their results.

    Our response: "It was corrected according to your recommendation."
    The results of this study, which reveal a high prevalence of dengue, underscore the need to implement a more comprehensive diagnostic approach in the different regions of Peru. In particular, our findings suggest the importance of using diagnostic tests with high sensitivity to detect acute infections, such as NS1 antigen detection tests, which can identify the virus in the early stages of infection. We also recommend the use of molecular tests, such as PCR, to improve diagnostic accuracy, especially in areas with high circulation of multiple dengue serotypes. Evaluating the sensitivity and specificity of these tools in various regions will help to optimize their use in local contexts.

If you have any comments or recommendations, we are ready to respond.

Sincerely,

Mario J. Valladares-Garrido

Universidad Continental, Lima 15046, Peru; mvalladares@continental.edu.pe
