# Supplementary material for: Prevalence of dengue in febrile patients in Peru: A systematic review and meta-analysis
Source: PLoS One. 2025 Jun 17;20(6):e0310163. doi: 10.1371/journal.pone.0310163 (PMC12173410; doi:10.1371/journal.pone.0310163)
Supplement: Attachment — Submitted filename: Response to Reviewers.pdf [file pone.0310163.s010.pdf]

Dear **Editor**,

Thank you very much for reviewing our article, " Prevalence of dengue in Peruvian patients with febrile illness according to RT-PCR and ELISA NS1, IgM, and IgG diagnostic tools: a systematic review and me-ta-analysis". Your suggestions and comments will be addressed below. Thank you for your valuable time and excellent review.

Editor's comments

1. The reviewers consider that your writing needs major modifications; it is very important that you clarify the biases that the study design may have when discussing dengue prevalence in Peru.
   Our response: Thank you very much for your comments, dear editor. The manuscript has been carefully revised for language, and each reviewer received a detailed response. All recommendations from the reviewers were accepted. Additionally, the meta-analysis was verified, and the article was reorganized in accordance with the PLOS ONE author guidelines.

Reviewer #2:

The authors refer both to the incidence of dengue and the prevalence of dengue within the text. As an acute infection when speaking of the disease itself it is correct to use incidence. I understand that due to the information the authors were reviewing positivity within a community (including positivity of serological test). I suggest talking of prevalence of positive dengue results instead of the prevalence of dengue or always clarify that you are talking of febrile patients, or prevalence of dengue antibodies if talking only of serological results. If the authors agree with this suggestion it would apply to:

• conclusion section of the abstract
• the outcomes section in material and methods
• the last paragraph of data analysis section in material and methods
• characteristics of the included studies section in results
• the Prevalence of dengue in Peruvian patients with febrile illness according to NS1, IgM, IgG ELISA, and RT-PCR section in results
• In discussion
• In Conclusions

Our response: Thank you for your insightful observation. We agree with your suggestion and have revised the manuscript accordingly to ensure consistency in the terminology. Specifically, we now refer to the "prevalence of positive dengue results" when discussing diagnostic outcomes in febrile patients and specify "prevalence of dengue antibodies" when referring exclusively to serological results. These clarifications have been applied throughout the relevant sections, including the abstract, methods, results, discussion, and conclusions. We appreciate your guidance, which helped enhance the precision and clarity of the manuscript.

1. **Reviewer says:** The authors might also want to mention that dengue has an endemic and epidemic behavior, so it is normal to see spikes in cases, and the number of cases fluctuates during the year making it hard to compare and contrast "prevalence".

   Our response: Thank you for your suggestion. We have revised the Discussion section to include an explanation of dengue's endemic and epidemic behavior. Specifically, we now highlight how seasonal fluctuations and outbreak periods contribute to variability

in case numbers and complicate direct comparisons of prevalence across studies. We also emphasize the importance of considering this epidemiological context when interpreting our findings. Your comment significantly enhanced the clarity and epidemiological accuracy of our analysis, and we are grateful for your contribution.

Reviewer #3:

Our response: Dear reviewer, we sincerely thank you for your valuable comments, which have greatly contributed to improving the quality of our manuscript. We have addressed each of your observations and suggestions, detailing the modifications made to the manuscript and providing a thorough response to each point raised.

1. **Reviewer says:** The goal of the study is to provide an overall estimate for dengue among those presenting with febrile illness (i.e., the meta-analysis should be relying on descriptive studies to produce results). LILLACS would represent a primary source of information for this and should have been a primary source when searching for manuscripts for this meta-analysis.
Our response: Thank you for your comment. Dear reviewer, we have utilized 8 databases and information sources, which are detailed in the search strategy.
We agree with your observation regarding LILLACS as a specialized database for scientific literature from Latin America and the Caribbean. However, the Virtual Health Library (VHL) integrates regional databases that are important for Latin America, which justifies its inclusion in our search strategy. We have attached evidence to support this decision.
We hope this clarifies your comment.

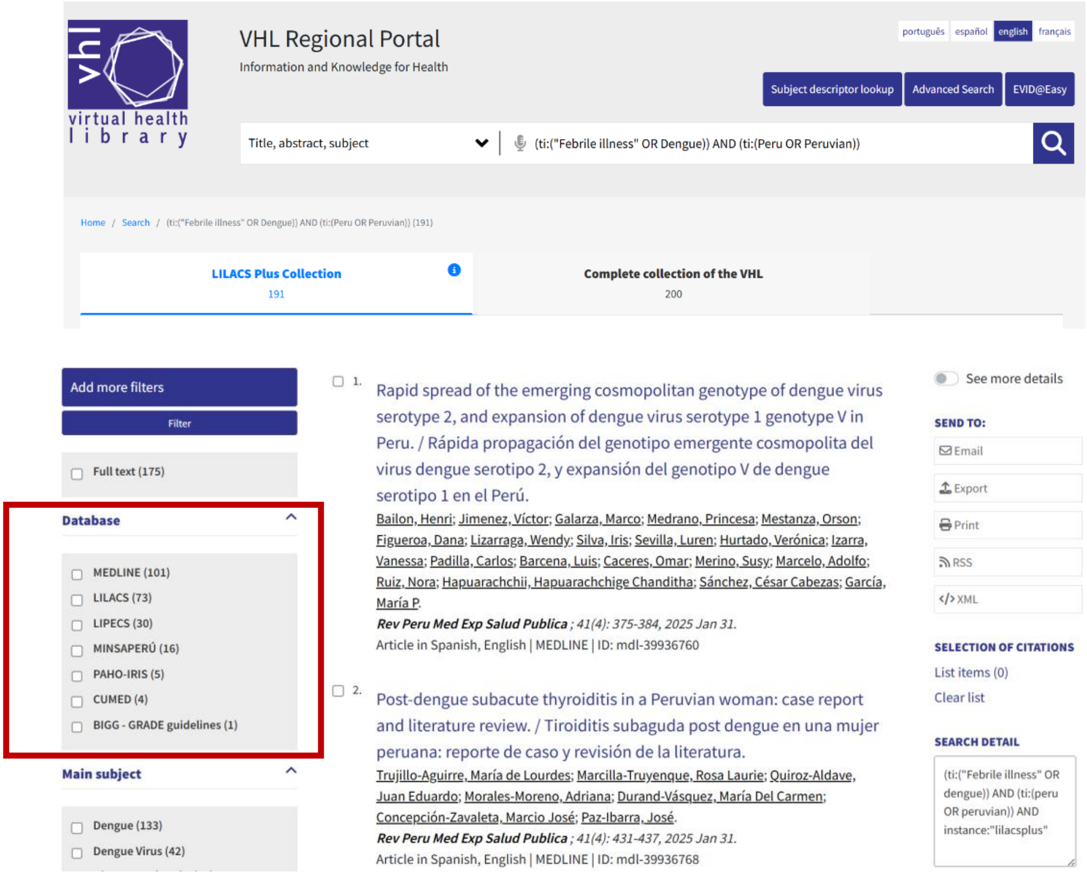

https://pesquisa.bvsalud.org/portal/?output=&lang=en&from=&sort=&format=&count=&fb=&page=1&tab=&skfp=&index=&q=%28ti%3A%28%22Febrile+illness%22+OR+Dengue%29%29+AND+%28ti%3A%28Peru+OR+Peruvian%29%29

2. **Reviewer says:** Instead, the analysis has selectively relied on 15 studies, including those describing outbreak investigations (ref: 34, 37), observational studies (ref: 35), and technology assessments of diagnostic tests (ref: 18, 30). None of these studies are designed to provide valid disease prevalence assessments, and, if used for that purpose could provide incorrect estimates. At the same time, previously published descriptive studies specifically designed to answer the research question of this meta-analysis (i.e., prevalence of dengue among those reporting febrile illness), using the same dengue diagnostic tests as those included in this meta-analysis, appear to have been excluded with the reason "No diagnostic test for dengue" (e.g., Forshey BM et al. PLOS Negl Trop Dis. 4(8): e787). It is also unclear why the LILLACS database was not included in the search for manuscripts.
   Our response: Thank you for this observation. It is true that some of the studies selected in our meta-analysis are more informative regarding outbreaks and the evaluation of diagnostic technologies. However, these studies were chosen because of their relevance in terms of the diagnostic methodologies used and the population of interest (fever patients). Although they were not specifically designed to assess disease prevalence, many of them provide valuable information on prevalence in febrile populations suspected of dengue. For the descriptive studies that were excluded, the main reason was the lack of validated diagnostic tests in these studies, which would have compromised the quality of prevalence estimates.
   Our systematic review has been conducted in accordance with PRISMA guidelines, following a rigorous methodology. Clearly defined eligibility criteria have been applied, and the feedback received has been constructive.
   Regarding LILACS, this has been addressed in the previous observation. Our research covers databases and information search engines at local, regional, and international levels. We provide a link for you to explore the virtual health library.

3. **Reviewer says:** There is also no discussion on issues of geographic and seasonal differences in dengue or recent outbreaks of the disease and government responses to control it. (e.g., Puno is not considered a high-risk area for dengue and the disease appears more often during the rainy season.) The manuscript does not discuss government surveillance estimates for the disease (i.e., CDC MINSA) or public health efforts to control the disease in the midst of recent outbreaks. The manuscript also covers the time-period of COVID-19. How could this have impacted results?
   Our response:
   We appreciate this comment. It is important to acknowledge the geographic and seasonal variability in dengue prevalence, which influences the results. We have expanded the discussion to consider geographic variations, where dengue is not considered a high-risk area but can appear during the rainy season. Furthermore, we have incorporated a discussion on government responses, including vector control measures and public health surveillance efforts. The influence of COVID-19 has also been addressed, recognizing that the pandemic may have affected the rates of diagnosis and reporting, given the disruptions in healthcare services and prioritization of other health concerns.

4. **Reviewer says:** Editor's & Reviewer Comments not addressed

Our response: Dear reviewer, the observations made by the initial reviewers have been addressed, as well as those from the editor. We are grateful for your constructive recommendations.

5. **Reviewer says:** - Data Availability Statement (Editor comment): Data on the evaluation of manuscripts for bias. The manuscript provides boilerplate language to the field of epidemiology, but no specifics are given as to what is specifically being evaluated (e.g., why is confounding important to evaluate if this is a descriptive study?).
   Our response: Thank you for pointing this out. In the "Data Availability Statement," we have revised the text to provide more detail about how we evaluate potential biases, including an explanation of why confounding factors are important to consider, even in descriptive studies. Although this study is descriptive, it is crucial to account for confounders such as geographical differences, seasonal variations, and access to healthcare services, which may affect the results. This clarification has been added to the study quality section to better explain how confounding factors can impact the interpretation of the findings.

6. **Reviewer says:** - Search of LILLACS for manuscripts (Editor & Reviewer #1 comment): It is still unclear why it was excluded from the analysis.
   Our response:
   Dear reviewer, this observation has been raised again significantly, but it has already been adequately addressed in our previous response. In a systematic review, it is common to use multiple databases. While we understand and agree with your emphasis on using LILACS, we have chosen to use a platform that includes LILACS: the Virtual Health Library (VHL), which is regional and highly important within Latin America. This platform provides access to a wide range of resources relevant to our research.
   We appreciate your understanding and valuable recommendations.

7. **Reviewer says:** - Need to include the statement on observational studies (Reviewer #1): There continues to be a confusion regarding what a descriptive study and observational design are investigating.
   Our response: This is a key point, and we thank you for bringing it up. We have reviewed the text to clarify the distinction between descriptive and observational studies. A descriptive study aims to identify characteristics of a population without intervention, while observational studies, although also non-interventional, seek to associate variables or explore relationships between them. We have added a more detailed explanation of these concepts in the introduction and methodology sections to avoid confusion.

8. **Reviewer says:** - Limitations of the work (Reviewer #1): The manuscript does not address key issues related to geographic and spatial heterogeneity of dengue estimates as well as changes in prevalence estimates during COVID-19.
   Our response: Thank you for your observation. We have expanded the discussion to include geographic and spatial heterogeneity in prevalence estimates, especially in regions like Piura, Lambayeque, and other northern areas of Peru, which are influenced by climatic factors such as the El Niño phenomenon. Additionally, we have included a specific section on the potential impact of COVID-19 on prevalence estimates, considering how healthcare disruptions and changes in the clinical presentation of febrile illnesses could have affected diagnostic rates.

9. **Reviewer says:** - Implication of study findings (Reviewer #2): There is no discussion of these results in relation to government public health efforts to control dengue or even current government surveillance efforts to track the disease.

   Our response: Thank you for pointing out this omission. We have added a section discussing the implications of our findings in relation to government efforts to control dengue, including vector control strategies, prevention campaigns, and surveillance systems such as those provided by the CDC and MINSA in Peru. This section has also been enriched with an analysis of public health policies and their interaction with recent outbreaks, providing a more comprehensive view of the current situation.

Reviewer #4:

This study aims to assess the prevalence of dengue virus in febrile patients in Peru. Notable strengths of the study include a well-designed methodology and a robust study population. However, certain minor aspects could be refined to further enhance the quality of the presentation. It is recommended that the authors take these into consideration. It is recommended that the text be edited for better readability.
Our response: We sincerely thank Reviewer [4] for the careful review of our manuscript and for the constructive feedback provided. We greatly appreciate your thoughtful observations and suggestions, which have helped us to improve the quality and clarity of our work. All of your comments have been addressed in detail, and the corresponding changes have been incorporated into the revised manuscript accordingly.

1. **Reviewer says:** 1- Suggested shorter title: "Dengue Prevalence in Febrile Patients from Peru: A Systematic Review and Meta-Analysis"
   Our response: Thank you for your thoughtful suggestion. We agree that the proposed title is clearer and more concise. Accordingly, we have revised the manuscript title to: "Prevalence of dengue in febrile patients in Peru: a systematic review and me-ta-analysis."

2. **Reviewer says:** 2- Mentioning diagnostic methods in the study background is unnecessary and should be removed. In the abstract avoid including unnecessary details such as: "Study selection, quality assessment, and data extraction were performed independently by more than two authors." and "The study protocol was registered in PROSPERO (CRD4202424558891)." Simply mentioning the methods is sufficient and enhances the readability of the abstract.
   Our response: We appreciate your valuable comments and have made the corresponding revisions. In the Background section, we removed the mention of diagnostic methods (RT-PCR and ELISA for NS1, IgM, and IgG), as they were not essential to the overall framing of the study. Additionally, in the abstract, we eliminated the methodological details deemed unnecessary, specifically the phrases "Study selection, quality assessment, and data extraction were performed independently by more than two authors" and "The study protocol was registered in PROSPERO (CRD4202424558891)," in order to enhance the clarity and readability of the text, as suggested. We thank you again for your helpful input in strengthening our manuscript.

3. **Reviewer says:** 3- The introduction is well-written and provides valuable insights into the significance and characteristics of the disease, as well as its regional status in Peru.

However, some sections could be more concise to enhance the flow and improve transitions between topics. It is recommended to shorten the introduction for better readability.

Our response: We appreciate your thoughtful and constructive feedback. In response to your suggestion, we have revised the Introduction to improve its conciseness and flow. Specifically, we shortened detailed sections—particularly those related to diagnostic methods and immunopathological mechanisms—which were more appropriate for the Methods or Discussion sections. We also enhanced the transitions between paragraphs to ensure a clearer narrative. Thank you again for your valuable comments, which helped strengthen the clarity and readability of our manuscript.

4. **Reviewer says:** 4- "The original protocol registered in PROSPERO was modified with minor adjustments." Clearly define what these "minor adjustments" entailed and provide a rationale for each change.
   Our response: Thank you for your observation. In response, we have revised the manuscript to clearly define the minor adjustments made to the original PROSPERO-registered protocol. These included: (1) expanding the inclusion criteria to incorporate studies with and without control groups, allowing for a more comprehensive analysis; (2) updating the list of authors to reflect changes in team participation; and (3) including additional researchers in the screening, evaluation, and data extraction processes to strengthen methodological rigor and reduce potential bias. We have also explained the rationale for each adjustment in the revised section. These changes were implemented prior to data analysis and did not alter the study's primary objectives or methodology. We appreciate your suggestion, which helped improve the clarity and transparency of our reporting.

5. **Reviewer says:** 5- "During a meeting, the three independent authors compared their data extractions and reached a consensus to resolve any discrepancies." Clarify how consensus was achieved—whether through discussion, statistical methods, or another approach.
   Our response: Thank you for your helpful suggestion. In accordance with your recommendation, we have revised the manuscript to clarify how consensus was achieved among the three independent authors during the data extraction process. Specifically, we now explain that consensus was reached through structured discussion, in which each author presented their rationale, and discrepancies were resolved by jointly reviewing the full text of the articles to ensure accuracy. This clarification has been incorporated into the revised version of the section. We appreciate your input, which helped improve the transparency and methodological rigor of our work.

If you have any comments or recommendations, we are ready to respond.

Sincerely,

Mario J. Valladares-Garrido

Escuela de Medicina Humana, Universidad Señor de Sipán, Chiclayo, Peru; vgarrido@uss.edu.pe (M.J.V.G)
