# Supplementary material for: Prevalence of dengue in febrile patients in Peru: A systematic review and meta-analysis
Source: PLoS One. 2025 Jun 17;20(6):e0310163. doi: 10.1371/journal.pone.0310163 (PMC12173410; doi:10.1371/journal.pone.0310163)
Supplement: Attachment — Submitted filename: Response_to_Reviewers_auresp_3.pdf [file pone.0310163.s011.pdf]

Dear **Editor**,

Thank you very much for reviewing our article, " Prevalence of dengue in febrile patients in Peru: a systematic review and meta-analysis". Your suggestions and comments will be addressed below. Thank you for your valuable time and excellent review.

**Editor's comments**

1. Please review your reference list to ensure that it is complete and correct. If you have cited papers that have been retracted, please include the rationale for doing so in the manuscript text, or remove these references and replace them with relevant current references. Any changes to the reference list should be mentioned in the rebuttal letter that accompanies your revised manuscript. If you need to cite a retracted article, indicate the article's retracted status in the References list and also include a citation and full reference for the retraction notice.
   **Our response:** Thank you for your comment. We have carefully reviewed the reference list to ensure it complies with the citation guidelines of PLOS One. After a thorough review, we can confirm that all cited references are accurate and do not include any retracted articles.

**Reviewer #2:**

We deeply appreciate your comments and recommendations, which have been instrumental in enhancing the quality of our article. Below, we will provide a detailed response to each of the suggestions given.

1. The paper has improved greatly, I believe it holds interesting findings on an important topic.
   **Our response:** Thank you very much for your positive feedback. We are glad to hear that you find the paper's improvements and its findings on this important topic valuable. Your comments have been instrumental in refining our work, and we appreciate your support throughout the process.

2. I still feel that two minor things should be addressed, one regarding the prevalence of an acute disease in febrile patients through IgG that can persist for months or years after the infection. This was improved from previous iterations of the manuscript, and with a mention of this in the limitations section.
   **Our response:** Thank you for your comment. We have addressed this concern by ensuring clarity regarding the use of IgG as a marker for past infection rather than acute disease. In the manuscript, we have replaced references to IgG with the term "dengue antibodies," as it is important to distinguish between antibodies from past infections and active disease. We have made these changes in the abstract, results, and discussion sections to more accurately reflect the diagnostic role of IgG. Additionally, we have included a mention in the limitations section that IgG may not represent current infection and could reflect past exposure. We hope this revision provides the necessary clarification.

3. Second, I think the authors should clarify the concept of studies having controls within their search strategy.
   **Our response:** Thank you for pointing this out. We have revised the manuscript to clarify the concept of control groups in our study selection process. Specifically, we have stated that the control group consisted of febrile patients who did not have dengue infection. These revisions have been added in the Protocol and Registration

section, as well as the Eligibility Criteria section, to ensure consistency and reduce confusion.

4. I still believe the authors should be more guarded when they talk about the prevalence of dengue through IgG ELISA, even in a febrile patient; unless those patients had an associated positive PCR result, or at least a positive NS1 result, IgG is not a diagnostic test, and thus the authors should talk about the prevalence of dengue antibodies when reference those results. For example, in the 3rd line of the results section of the abstract, the authors could talk about the "…prevalence of dengue or dengue antibodies…".
   **Our response:** We appreciate your thorough review. We have revised the manuscript to reflect a more cautious approach regarding the interpretation of IgG ELISA results. The term "dengue antibodies" has been consistently used throughout the manuscript to avoid any confusion that IgG is being used as a diagnostic marker for active infection. In the abstract, results section, and discussion, we now refer to "the prevalence of dengue or dengue antibodies" rather than stating a direct prevalence of dengue infection. We believe this modification aligns better with the limitations of IgG as a diagnostic tool.

5. This is seen again throughout the results, like in section 3.3 and parts of the discussion. This issue has been addressed and corrected in other sections, like the first two sentences of the discussion section.
   **Our response:** Thank you very much for your observation. The entire article was reviewed and corrected to standardize the terminology.

6. In the second sentence of the 5th and last paragraph of the introduction, I would review redaction, avoiding the repetition of the word rapid.
   **Our response:** Corrected

7. In the materials and methods section in the Protocol Registration section, the authors mention that "inclusion criteria were expanded to allow studies both with and without a control group". However, in the Eligibility Criteria section, they still include that "investigation included a control group", followed by "Studies without a control group were included" in the very next sentence, making this a contradiction within the eligibility criteria. I suggest framing this in a similar way to the one used in the Protocol Registration section.
   **Our response:** Thank you for pointing out this contradiction. We have revised the Eligibility Criteria section to align the language with the Protocol and Registration section. The revised text now states: "The control group was composed of febrile patients who did not have dengue infection, while the non-control group consisted of febrile patients who tested positive for dengue." This change ensures consistency and eliminates any confusion.

8. Moreover, I would clarify this because febrile patients with negative results for dengue are not a control group, as this is a result of a test outcome and not a predetermined group in the study design. Unless you are talking of patients with confirmatory laboratory criteria (PCR or NS1) as control and disease groups to evaluate the performance of non-confirmatory laboratory tests (single sample IgM).
   **Our response:** We appreciate your observation. To clarify, we have revised the manuscript to emphasize that the control group refers to febrile patients with negative dengue test results, specifically those who did not have dengue infection.

We have also stated that studies using febrile patients with negative dengue test results are considered controls only if their absence of infection was confirmed through diagnostic testing (e.g., PCR or NS1). This ensures that the inclusion of febrile patients with negative results is appropriately framed.

9. Looking at table 1, under Study type, non of the studies were case control studies.
   In the methods, in the Data collection process and data items section, I suggest changing the wording for "the region of development", maybe something as simple as study site, or geographical region, might work better.
   **Our response:** Thank you very much for your comment. Table 1 was reviewed, specifically in the section related to the type of study. Additionally, in the Methods section, under 'Data collection process and data elements,' the wording was changed from 'region of development' to 'geographic region'.

**Reviewer #4:**

All comments have been addressed

**Our response:** We deeply appreciate the valuable recommendations and observations you provided. Thanks to your feedback, our article has undergone a substantial improvement in terms of quality, clarity, and academic rigor. Your contributions have been essential in enriching the content and strengthening the foundation of our research.

If you have any comments or recommendations, we are ready to respond.

Sincerely,

**Mario J. Valladares-Garrido**

Escuela de Medicina Humana, Universidad Señor de Sipán, Chiclayo, Peru; vgarrido@uss.edu.pe (M.J.V.G)
